# High rates of submicroscopic aberrations in karyotypically normal acute lymphoblastic leukemia

**DOI:** 10.1186/s13039-015-0153-4

**Published:** 2015-06-30

**Authors:** Moneeb A. K. Othman, Joana B. Melo, Isabel M. Carreira, Martina Rincic, Anita Glaser, Beata Grygalewicz, Bernd Gruhn, Kathleen Wilhelm, Katharina Rittscher, Britta Meyer, Maria Luiza Macedo Silva, Terezinha de Jesus Marques Salles, Thomas Liehr

**Affiliations:** Jena University Hospital, Friedrich Schiller University, Institute of Human Genetics, Jena, Germany; Laboratory of Cytogenetics and Genomics, Faculty of Medicine, University of Coimbra, Coimbra, Portugal; CIMAGO, Centro de Investigação em Meio Ambiente, Genéticae Oncobiologia, Coimbra, Portugal; Croatian Institute of Brain Research, Zagreb, Croatia; Cytogenetic Laboratory, Maria Sklodowska-Curie Memorial Cancer Centre and Institute, Warsaw, Poland; Department of Pediatrics (Oncology and Hematology), Jena University Hospital, Friedrich Schiller University, Jena, Germany; ZytoVision GmbH, Bremerhaven, Germany; Cytogenetics Department, Bone Marrow Transplantation Unit, National Cancer Institute, Rio de Janeiro, RJ Brazil; Post Graduation Program in Oncology, National Cancer Institute (INCA), Rio de Janeiro, RJ Brazil; Pediatric Oncohematology Center, Hospital Oswaldo Cruz/ Pos Graduation Course of the Faculty of Medical Sciences, University of Pernambuco, Recife, PE Brazil

**Keywords:** Multitude multicolor banding (mMCB), Acute lymphoblastic leukemia (ALL), Cryptic rearrangements, Fluorescence *in situ* hybridization (FISH), Multiplex ligation-dependent probe amplification (MLPA), Array-comparative genomic hybridization (aCGH)

## Abstract

**Background:**

Acute lymphoblastic leukemia (ALL) is not a single uniform disease. It consists of several subgroups with different cytogenetic and molecular genetic aberrations, clinical presentations and outcomes. Banding cytogenetics plays a pivotal role in the detection of recurrent chromosomal rearrangements and is the starting point of genetic analysis in ALL, still. Nowadays, molecular (cyto)genetic tools provide substantially to identify previously non-detectable, so-called cryptic chromosomal aberrations in ALL. However, ALL according to banding cytogenetics with normal karyotype - in short cytogenetically normal ALL (CN-ALL) - represent up to ~50 % of all new diagnosed ALL cases. The overall goal of this study was to identify and characterize the rate of cryptic alterations in CN-ALL and to rule out if one single routine approach may be sufficient to detect most of the cryptic alterations present.

**Results:**

Sixty-one ALL patients with CN-ALL were introduced in this study. All of them underwent high resolution fluorescence *in situ* hybridization (FISH) analysis. Also DNA could be extracted from 34 ALL samples. These DNA-samples were studied using a commercially available MLPA (multiplex ligation-dependent probe amplification) probe set directed against 37 loci in hematological malignancies and/or array-comparative genomic hybridization (aCGH). Chromosomal aberrations were detected in 21 of 61 samples (~34 %) applying FISH approaches: structural abnormalities were present in 15 cases and even numerical ones were identified in 6 cases. Applying molecular approaches copy number alterations (CNAs) were detected in 27/34 samples. Overall, 126 CNAs were identified and only 34 of them were detectable by MLPA (~27 %). Loss of CNs was identified in ~80 % while gain of CNs was present in ~20 % of the 126 CNAs. A maximum of 13 aberrations was detected per case; however, only one aberration per case was found in 8 of all in detail studied 34 cases. Of special interest among the detected CNAs are the following new findings: del(15)(q26.1q26.1) including *CHD2* gene was found in 20 % of the studied ALL cases, dup(18)(q21.2q21.2) with the *DCC* gene was present in 9 % of the cases, and the *CDK6* gene in 7q21.2 was deleted in 12 % of the here in detail studied ALL cases.

**Conclusions:**

In conclusion, high resolution molecular cytogenetic tools and molecular approaches like MLPA and aCGH need to be combined in a cost-efficient way, to identify disease and progression causing alterations in ALL, as majority of them are cryptic in banding cytogenetic analyses.

**Electronic supplementary material:**

The online version of this article (doi:10.1186/s13039-015-0153-4) contains supplementary material, which is available to authorized users.

## Background

Acute lymphoblastic leukemia (ALL) is a malignant disease of the hematological system with clonal proliferation of lymphoid progenitor cells. It arises from genetic alterations that block precursor B and T cell differentiation and predominantly affects children [1]. B-ALL constitutes 80-85 % of ALL cases and T-ALL the remainder ones. B-ALL patients have a favorable prognosis with an overall complete remission rate of 95 % for pediatric (children and adolescent between 1–15 years) but of only 60 % for adults. Adverse prognosis in T-ALL was correlated with presence of hyperleukocytosis, enhanced mediastinal mass, central neural system involvement, male gender and advanced age [1–5]. Cytogenetically detectable structural or numerical chromosomal abnormalities are detected in ~50 % of ALL cases. Such aberrations have prognostic significance [1, 6]. High hyperdiploidy (51–65 chromosomes) has been connected with good survival and excellent outcome in B-ALL, while hypodiploidy (<44 chromosomes) has an adverse prognosis [7–9]. Recurrent structural chromosomal abnormalities found in ALL can also be reciprocal translocations. ALLs with a translocation t(12;21)(p13;q22) leading to the *ETV6/RUNX1* gene-fusion are more likely to be cured, than those with a translocation t(9;22) or t(4;11), which tend to have unfavorable outcomes. Complex karyotypes, including three to five or more chromosomal abnormalities, are typically found in ~5 % of ALL cases and are also associated with an adverse outcome [10]. Finally, ALL cases with according to banding cytogenetics normal karyotype - in short cytogenetically normal ALL (CN-ALL) - are classified into intermediate risk group [6, 11, 12]. Malignant bone marrow of T-ALL patients shows a normal karyotype more frequently than those of B-ALL patients. Accordingly in those cases cytogenetic markers cannot be determined and therapeutic decisions may be hampered.

Based on the knowledge that chromosomes in ALL show a low banding resolution and that a good part of ALL cases present with a normal karyotype, it is not far to seek, that small aberration can easily be missed when analyzing ALL derived chromosomes by banding cytogenetics alone [6, 13]. Copy number alterations (CNAs) at the microscopic or submicroscopic level, i.e. focal deletions, but also duplications or sequence/point mutations in genes that primarily serve as transcriptional regulators of the lymphoid developmental pathway can nowadays be detected by approaches like multiplex ligation-dependent probe amplification (MLPA) or array-comparative genomic hybridization (aCGH) [12, 14, 15].

The present study includes 61 CN-ALL cases, which were retrospectively studied for the rate of cryptic (sub)chromosomal changes to rule out if one single molecular (cyto)genetic routine approach may be sufficient to detect most if not all of the cryptic alterations present.

## Results

**Figure 1:**
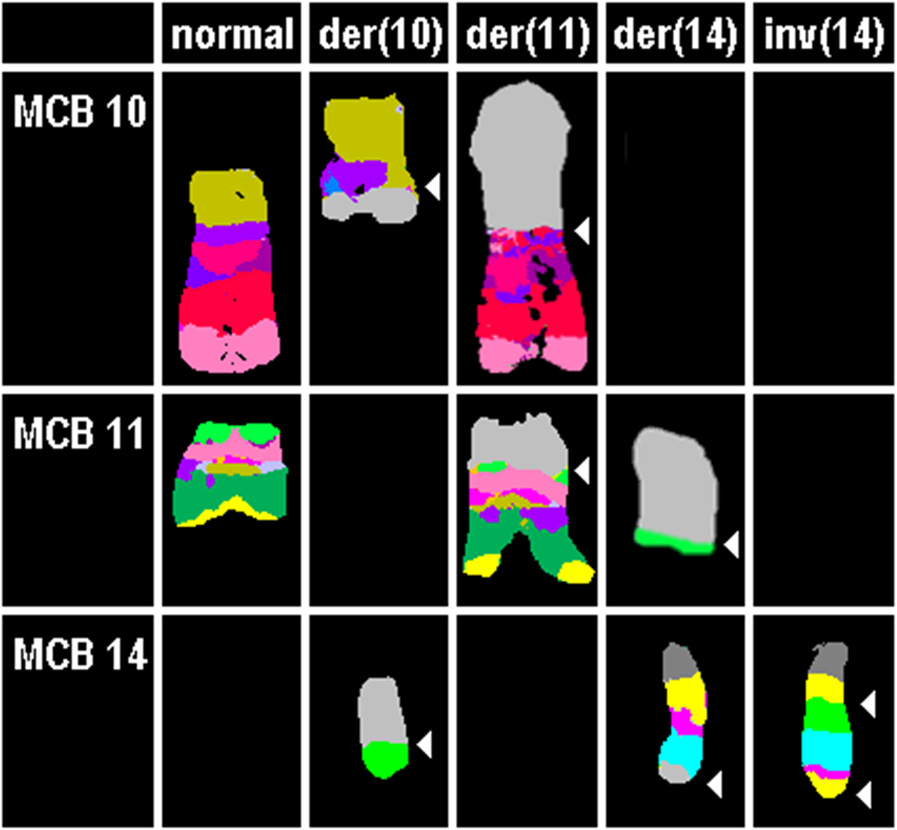
Result of aMCB probesets for chromosomes 10, 11, and 14 are shown, which characterized the breakpoints seen in case P66 as 10q11.23, 11p15.3, 14q11, 14q24.2, and 14q32.3. The final karyotype after application of all approaches as summarized in Additional file 1: Table S1 was 46,XX,der(10)(10pter- > 10p12.31::11q23.3- > 11q23.3::10p12.31- > 10q11.23::14q24.2- > 14qter),der(11)(10qter- > 10q11.23::11p15.3- > 11q23.3::10p12.31- > 10p12.31::11q23.3- > 11qter),der(14)t(11;14)(q15.3;q24.2),inv(14)(q11q23)

Standard cytogenetic analysis by G-banding revealed normal karyotypes in 61 ALL cases included in this study (Additional file 1: Table S1). In a first step all 61 cases were studied by the whole genome oriented fluorescence *in situ* hybridization (FISH)-banding based probe set multitude multicolor banding (mMCB) [16]. For further delineation of mMCB results appropriate FISH-probes and probe sets were applied (Additional file 1: Table S1). Based on these results 21/61 (34 %) cases were not cytogenetically normal but had gross acquired chromosomal aberrations: structural abnormalities were found in 15/61 cases (24 %) and even numerical ones were observed in 6/61 cases (10 %) (Table 1). Overall, in GTG-banding cryptic balanced and unbalanced translocations, derivative chromosomes, isochromosomes, interstitial deletions, inverted duplications and/or numerical aberrations were identified in 34 % of the studied CN-ALL cases by means of molecular cytogenetics. In Fig. 1 case P66 is exemplified with a three-way translocation between chromosomes #10, #11 and #14, inversion of second chromosome # 14 and insertion (11;10). The breakpoints of this P66 case were characterized as 10p12.3, 10q11.23, 11p15.3, 11q23.3, 14q11, 14q24.2, and 14q32.3.

**Table 1:** Summary of aberrations detected by metaphase directed FISH, interphase FISH to determine the percentage of specific aberrations, and aCGH in 34 ALL patients

Case number	Age [y]	Metaphase directed FISH	MLPA	LSPs for genes	aCGH – affected cytobands	Localization acc. to GRCH37/hg19	Size of imbalance [bp]
B-ALLs							
P1	1	46,XX	normal	normal	dup(11)(p15.5p15.4)	chr11:1,960,555-3,626,932	1,666,377
P8	30	47,XY,+21[5]/46,XY[2]	dup of 21q22.12	*RUNX1*: dup (72 %)	n.d.	n.d.	n.d.
P13	34	46,XY[8]	del of 10q23.3		del(10)(q23.2q23.31)	chr10:88,906,902-91,189,599	2,282,697
			del of 17p13.1	*TP53*: del (9 %)	del(17)(p13.1p13.1)	chr17:7,579,695-8,281,928	702,233
P17	27	46,XX[7]	n.d.	normal	normal	n.d.	n.d.
P23	59				del(3)(p25.3p25.3)	chr3:10,179,706-10,385,195	205,489
			del of 7p12.2		del(7)(p12.2p12.2)	chr7:50,337,405-50,482,274	144,869
					del(10)(q23.3q23.3)	chr10:89,570,600-89,676,741	106,141
					del(11)(q14.2q14.2)	chr11:85,683,188-85,944,362	261,174
		47,XX,+14[2]/		*IGH*: dup (58 %)	+14	+14	107,349,540
		46,XX[3]			del(15)(q26.1q26.1)	chr15:93,390,484-93,463,312	72,828
					del(17)(p13.1p13.1)	chr17:7,581,198-7,922,308	341,110
					del(17)(q11.2q11.2)	chr17:30,259,053-30,271,653	12,600
					del(18)(q21.32q21.32)	chr18:57,517,756-57,718,190	200,434
					del(21)(q22.3q22.3)	chr21:45,527,941-45,565,198	37,257
P28	84	46,XY,	del of 7p12.2		del(7)(p12.2p12.2)	chr7:50,353,062-50,444,269	91,207
		t(9;22)(q34;q11),	del of 9p21.3	*CDKN2A/B*: del (75 %)	del(9)(pterp11.2)	chr9:0–47,212,321	47,212,321
		del(11)(q13q25)[7]	del of 9p13.2		del(9)(q34.2qter)	chr9:136,917,580-141,213,431	4,295,851
					del(10)(q23.3q23.3)	chr10:89,619,806-89,731,258	111,452
			del of 11q22.3	*BIRC3*: del (75 %)	del(11)(q13.2qter)	chr11:67,773,863-135,006,516	67,232,653
				*ATM*: del (77 %)	del(15)(q26.1q26.1)	chr15:93,412,860-93,450,773	37,913
				*MLL*: del (80 %)	dup(20)(q11.23q12)	chr20:37,305,876-39,130,131	1,824,255
					del(20)(q12q13.12)	chr20:39,245,111-45,524,952	6,279,841
					dup(20)(q13.12q13.12)	chr20:45,524,953-45,780,811	255,858
					del(20)(q13.12q13.32)	chr20: 45,780,812-58,067,678	12,286,866
					del(21)(q22.2q22.2)	chr21:39,764,621-39,807,169	42,548
				*BCR*: del (94 %)	del(22)(q11.23q11.23)	chr22:23,584,037-23,592,537	8500
P43	69	46,XX,	normal	*TFG*: dup (15 %)	dup(3)(q12.2q12.2)	chr3:100,360,682-100,444,109	83,427
		der(4)(4pter- > 4q21.3::11q23.3-			del(7)(q21.2q21.2)	chr7:92,252,341-92,475,197	222,856
		>11q23.3::4q21.3- > 4qter),		*MLL*: ins (75 %)			
		der(11)(11pter- > 11q23.3::11q23.3- > 11q24.2::11p15.4- > 11pter),					
		der(11)(11qter- > 11q24.2::11p15.4- > 11qter)[5]					
P48	39	46,XY,	n.d.		del(6)(q13q14.2)	chr6:73,331,571-84,140,938	10,809,367
		t(6;11)(q15;p12),			del(6)(q16.2q21)	chr6:99,282,580-109,703,762	10,421,182
		ins(6;11)(q22.1;q13q14),			del(6)(q22.31q22.33)	chr6:124,125,069-128,841,870	4,716,801
		inv(6)(q15q25.3),		*ESR1*: del (89 %)	del(6)(q25.1q25.3)	chr6:151,725,897-157,531,913	5,806,016
		del(11)(q21q23.2)[8]			del(7)(p12.2p12.2)	chr7:49,991,954-51,207,236	1,215,282
					dup(11)(p15.5p15.4)	chr11:1,925,114-3,143,116	1,218,002
				*WT1*: del (91 %)	del(11)(p15.1p12)	chr11:20,546,133-37,403,781	16,857,648
				*BIRC3*: del (90 %)	del(11)(q14.1q14.3)	chr11:85,157,088-88,557,421	3,400,333
				*ATM*: del (77 %)	del(11)(q22.1q22.3)	chr11:100,992,179-114,667,959	13,675,780
					del(13)(q14.2q14.2)	chr13:48,980,623-49,148,073	167,450
P49	39	46,XX[10]	n.d.	normal	dup(11)(p15.5p15.4)	chr11:2,016,406-3,430,378	3,430,378
P51	59	46,XX[6]	normal	normal	del(10)(p12.1p12.1)	chr10:28,057,099-28,220,314	163,215
					del(15)(q26.1q26.1)	chr15:93,412,860-93,450,773	37,913
					del(X)(q21.1q21.1)	chrX:76,875,639-77,157,819	282,180
P52	21	46,XY[4]		normal	del(6)(p21.1p21.1)	chr6:45,395,872-45,409,919	14,047
					del(7)(q21.2q21.2)	chr7:92,149,393-92,495,958	346,565
			del of 10q23.3		del(10)(q23.3q23.3)	chr10:89,610,886-89,722,948	112,062
					del(11)(q14.2q14.2)	chr11:85,683,188-85,944,362	261,174
					del(15)(q26.1q26.1))	chr15:93,433,130-93,450,773	17,643
					del(17)(q23.1q23.1)	chr17:57,698,768-57,913,528	214,760
					del(20)(q13.2q13.2)	chr20:52,151,411-52,629,609	478,198
					del(X)(p22.33p22.33)	chrX:1,327,561-1,684,270	1,684,270
P53	34	46,XY[5]	normal	normal	dup(22)(q11.21q11.21)	chr22:18,706,001-21,561,514	2,855,514
P55	19	46,XY[6]	del of 17p13.1	*TP53*: del (100 %)	del(17)(pterp11.2)	chr17:0–20,219,464	20,219,464
					−20	−20	63,025,520
P56	47	45,XY,-21[2]/	normal	normal	del(12)(pterp11.21)	chr12:0–31,260,891	31,260,891
		46,XY[4]					
P57	56	46,XY[3]	normal	normal	normal	n.d.	n.d.
P58	20	46,XX,		*TBL1XR1:* del (68 %)	del(3)(q26.32q26.32)	chr3:176,825,586-177,697,157	871,571
		der(14)(pter- > q32::q32- > q13::q32- > qter)[10]	del of 9p21.3	*CDKN2A/B*: del (74 %)	del(9)(p21.3p21.3)	chr9:21,252,517-24,289,720	3,037,203
					del(10)(p15.3p15.3)	chr10:1,491,986-1,582,072	90,086
				*IGH*: split (78 %)	dup(14)(q13q32.33)	chr14:35,918,265-106,513,022	70,594,757
					del(16)(q13q13)	chr16:57,275,940-57,331,138	55,198
					del(21)(q22.2q22.2)	chr21:39,764,621-39,895,171	130,550
P64	5	46,XX,	n.d.		del(5)(q31.3q32)	chr5:142,096,863-145,891,069	3,794,206
		t(16;19)(p11.2;q13.3),					
		der(5)t(5;9)(q31;p13.2),		*CDKN2A/B*: del (86 %)	del(9)(p21.3p21.3)	chr9:21,218,548-23,002,377	1,783,829
		der(9)t(5;9)(q31;p13.2),					
		der(9)t(9;9)(q34;p13.2)[10]		*FUS*: split (75 %)			
P66	0.5	46,XX,	n.d.	*MLL*: split (70 %)	dup(11)(p15.5p15.4)	chr11:1,008,688-3,669,161	3,669,161
		der(10)(10pter- > 10p12.31::11q23.3- > 11q23.3::10p12.31- > 10q11.23::14q24.2- > 14qter),		*IGH*: inv (100 %)			
		der(11)(10qter- > 10q11.23::11p15.3- > 11q23.3::10p12.31- > 10p12.31::11q23.3- > 11qter),					
		der(14)t(11;14)(q15.3;q24.2), inv(14)(q11q23)[8]					
T-ALLs							
P5	22	46,XX[12]	normal	normal	normal	n.d.	n.d.
P6	16	47,XY,	normal	normal			
		+4,			+4	+4	191,154,276
		der(3)t(3;5)(p23;q31.1),					
		der(5)t(3;5)(p23;q35.3),					
		der(5)t(5;10)(q31.1;p12.3),					
		der(10)t(5;10)(q35.3;p12.3)[8]/					
		46,XY[13]					
P7	26	46,XY,	del of 9p21.3	*CDKN2A/B*: del (64 %)	del(9)(p21.3p21.3)	chr9:21,817,082-23,515,821	1,698,739
		t(2;9;18)(p23.2;p21.3;q21.33),	del of 13q14.2	*RB1*: del (25 %)	del(13)(q14.2q14.2)	chr13:48,982,463-49,062,316	79,853
		t(10;14)(q24;q11)[10]			del(16)(p13.3p13.3)	chr16:3,154,954-4,568,792	1,413,838
P18	36	46,XY[5]	dup of 18q21.2	*DCC*: dup (13 %)	n.d.	n.d.	n.d.
P32	27	47,XX,	del of 6q21		n.d.	n.d.	n.d.
		+21,	del of 6q27				
		t(10;14)(q24;q11),	del of 9p21.3	*CDKN2A/B*: del (89 %)			
		del(6)(q15q27)[6]	del of 12p13.2	*ETV6*: del (78 %)			
			del of 13q14.3	*DLEU1*: del (15 %)			
			dup of 21q22.1	*RUNX1*: dup (78 %)			
P35	40	46,XY,i(9)(q21.11)[2]			del(2)(q34q34)	chr2:213,811,279-214,150,984	339,705
					dup(7)(pterp14.1)	chr7:0–38,218,586	38,218,586
					del(7)(q21.2q21.2)	chr7:92,252,341-92,460,773	208,432
					del(7)(q36.3qter)	chr7:156,881,580-159,138,663	2,257,083
			del of 9p21.3	*CDKN2A/B*: del (92 %)	del(9)(pterp11.2)	chr9:0–47,212,321	47,212,321
			del of 9p13.2		dup(9)(q21.11qter)	chr9:71,035,265-141,213,431	70,178,166
					del(10)(q23.2q23.31)	chr10:89,570,600-89,728,844	158,244
					del(11)(q22.2q22.2)	chr11:102,106,046-102,529,831	423,785
					del(13)(q14.2q14.2)	chr13:49,004,123-49,122,923	118,800
					del(15)(q26.1q26.1)	chr15:93,390,484-93,466,292	75,808
					del(16)(p13.3p13.3)	chr16:3,808,951-3,839,782	30,831
					del(18)(q21.32q21.32)	chr18:57,517,756-57,617,796	100,040
					del(20)(q13.2q13.2)	chr20:52,151,411-52,574,928	423,517
P38	22	46,XY[3]	normal	normal	normal	n.d.	n.d.
P61	18	46,XX,der(2)t(2;7)(q37.3;q34), t(7;10)(q34;q24.1 ~ 25.1) [4]/			del(1)(p36.31p36.23)	chr1:5,958,728-7,238,618	1,279,890
					del(4)(p16.3p14)	chr4:3,072,509-38,882,925	35,810,416
		46,XX[3]	dup of 6q23.3	*MYB*: amp (90 %)	dup(6)(q23.3q23.3)	chr6:134,245,761-136,118,354	1,872,593
			del of 9p21.3	*CDKN2A/B*: del (88 %)	del(9)(p21.3p21.3)	chr9:21,252,517-23,002,377	1,749,860
				*ABL1*: amp (95 %)	dup(9)(q34.1q34.1)	chr9:133,658,293-134,092,544	434,251
				*FGFR2*: del (57 %)	del(10)(q25.1q26.3)	chr10:112,392,101-135,534,737	23,124,636
B- or T ALLs (not clinically well defined)							
P11	26	46,XY[8]	n.d.	normal	normal	n.d.	n.d.
P16	17	46,XX[7]			del(1)(q25.3q31.1)	chr1:184,771,633-185,825,795	1,054,162
					del(4)(p15.33p15.31)	chr4:12,322,760-18,779,457	6,456,697
					del(4)(q21.22q24)	chr4:82,992,997-106,476,929	23,483,932
					del(7)(pterp14.2)	chr7:0–36,320,986	36,320,986
			dup of 7q22.1	*RELN*: dup (61 %)	dup(7)(q21.3q22.3)	chr7:96,048,870-106,348,693	10,299,823
					del(9)(p23p22.2)	chr9:12,656,733-17,466,907	4,810,174
			del of 9p21.3	*CDKN2A/B*: del (81 %)	del(9)(p21.3p21.3)	chr9:20,279,653-22,555,566	2,275,913
					del(10)(p14p13)	chr10:6,889,266-12,484,159	5,594,893
			del of 12p13.2	*ETV6*: del (91 %)	del(12)(p13.2p13.1)	chr12:11,761,018-12,934,870	1,173,852
					del(18)(p11.32p11.31)	chr18:2,741,687-3,231,531	489,844
P21	62	46,XY[11]	n.d.	normal	normal	normal	normal
P24	23	46,XY[12]	dup of 18q21.2	*DCC*: dup (18 %)	n.d.	n.d.	n.d.
P30	46	46,XY[6]	normal	normal	n.d.	n.d.	n.d.
P33	76	45,X,-X[8]			del(4)(q24q24)	chr4:106,036,993-106,601,946	564,953
					del(7)(q21.2q21.2)	chr7:92,080,855-92,475,197	394,342
					dup(7)(q36.2q36.2)	chr7:153,039,830-154,467,634	1,427,804
			del of 10q23.3		del(10)(q23.3q23.3)	chr10:89,610,886-89,698,312	87,426
					del(15)(q21.2q21.2)	chr15:51,826,924-51,919,665	92,741
					del(15)(q26.1q26.1)	chr15:93,433,130-93,450,773	17,643
			del of 17p13.1	*TP53*: del (10 %)	del(17)(p13.1p13.1)	chr17:7,583,457-8,156,734	573,277
					del(17)(q11.2q11.2)	chr17:30,259,193-30,267,204	8011
			dup of 18q21.2	*DCC*: dup (10 %)	dup(18)(q21.2q21.2)	chr18:49,105,579-51,431,815	2,326,236
					del(20)(q13.2q13.2)	chr20:52,151,411-52,554,455	403,044
					del(21)(q22.12q22.12)	chr21:36,253,465-36,426,708	173,243
					-X	-X	155,270,560
P46	63	46,XY[8]		normal	dup(6)(q25.3q25.3)	chr6:157,944,961-158,033,908	88,947
			del of 7p12.2		del(7)(p12.2p12.2)	chr7:50,452,798-50,492,798	40,000
					dup(17)(q12q12)	chr17:36,046,040-36,095,204	49,164
P47	59	46,XX[6]		normal	dup(1)(p13.3p13.3)	chr1:107,921,895-107,970,781	48,886
			del of 7p12.2		del(7)(p12.2p12.2)	chr7:50,356,873-50,465,376	408,503
			del of 9p13.2		del(9)(p13.2p13.2)	chr9:37,006,073-37,320,759	314,686
					dup(9)(q31.1q31.1)	chr9:104,126,808-104,167,077	40,269
					del(15)(q26.1q26.1)	chr15:93,390,484-93,450,773	60,289
					del(18)(q21.32q21.32)	chr18:57,517,756-57,718,190	200,434
					del(19)(p13.3p13.3)	chr19:0–2,787,457	2,787,457

*bp* basepairs, *LSP* locus-specific probes as specified in Additional file 2: Table S2, *y* year

**Figure 2:**
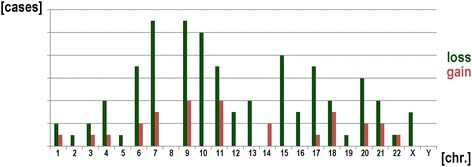
Distribution of CNAs as detected by aCGH in 27/34 studied cases. On X-axis the chromosome number is shown, while on Y-axis the total number of CNAs for each chromosome is depicted (scale 2).

34/61 studied CN-ALL cases (18 B-ALL, 8 T-ALL and 8 with undefined ALL) were studied further using MLPA and aCGH. Overall, 126 CNAs were detected by MLPA and aCGH in those cases. CNAs were identified in 27/34 (80 %) of the studied cases. 1 to 13 CNAs per case were detected (Table 1). The distribution of CNAs per chromosome and frequencies of gains and losses are summarized in Fig. 2; i.e. all chromosomes apart from 8 and Y were involved in CNAs in this study.

Deletions and duplications could be grouped according to their sizes as follows:

- focal CNAs (e.g. deletion of *CHD2* gene in 7 cases or duplication of *DCC* gene in 3 cases – Table 1);
- CNAs involving variable numbers of genes (e.g. deletion on 9p21.3 in 8 cases or amplification of 9q34.12q34.13 in one case – Table 1);
- CNAs involving large parts of whole chromosomal p and/or q arms (e.g. deletion on 4p16.3p14 in one case or duplication of 7p22.3p14.1 in one case – Table 1)
- CNAs of whole chromosomes (e.g. monosomy X in one case or trisomy #14 in one case – Table 1).

Most frequently observed deletion was 9p21.3 in 8/34 ALL cases (3x in B-ALL, 4x in T-ALL and 1x in undefined ALL); the *CDKN2A/B* genes were affected in all these eight cases. Furthermore, *PTEN* in 10q23.31 (6/34) and *IKZF1* in 7p12.2 (5/34) were the hit by deletions regularly. Besides, deletion in 15q26.1 (*CHD2* gene) was detected in 7/34 cases and duplication in 18q21.2 (*DCC* gene) in 3/34 cases.

## Conclusions

Cytogenetic analysis has been and still is the standard method for detection of diagnostically relevant recurrent chromosomal aberrations in ALL. It is well known that when using banding karyotyping cryptic chromosomal aberrations may be missed due to several reasons: (i) sensitivity of chromosomal banding techniques is limited, even in case of good chromosomal morphology, to aberrations being at least 10 Mb in size, (ii) aberrations may be cryptic or masked, i.e. they are not resolvable due to a similar or identical GTG-banding pattern and/or poor chromosome morphology, and (iii) metaphases may be difficult to obtain and to evaluated as chromosomes may not be well-spread, clumsy or appearing as fuzzy with indistinct margins; thus even numerical aberrations may be missed [6, 13, 17].

In the past molecular cytogenetic approaches have shown to be efficient to detect in banding cytogenetics cryptic chromosomal aberrations [6, 13, 17]. Besides in metaphase also interphase nuclei can be studied in case of low mitotic (non-dividing) cells and also alterations being at low mosaic level can be easily detected by that approach [12, 14, 18]. In this study, we detected previously cryptic aberrations in 21/61 (34 %) cases with ALL using metaphase directed FISH studies; even complex aberrations were identified in some of these cases (Table 1 and Additional file 1: Table S1).

For 34/61 cases DNA could be extracted from the cytogenetically worked up cell suspension. Thus, in those cases besides FISH also MLPA and aCGH could be applied additionally, i.e. approaches which have much higher resolution than FISH, but can only detect unbalanced aberrations and no low level mosaics. Using these approaches cryptic CNAs were detected in ~80 % of those ALL cases. All 126 CNAs detected by MLPA and aCGH have been checked by UCSC genome browser to exclude benign copy number variations (CNVs) (http://genome-euro.ucsc.edu/cgi-bin/hgGateway?redirect=auto&source=genome.ucsc.edu). Thus, all of them most likely are leukemia-related genetic changes, which were recognized in 27/34 ALL cases.

Of special interest may be a novel recurrent submicroscopic CNA expressed as loss of 15q26.1: focal deletion of *CHD2* gene located there was found in 7 of the 34 (20 %) studied ALL cases in this study. The *CHD2* gene is a member of the chromodomain helicase DNA-binding (CHD) protein family, which are all characterized by a chromatin-remodeling domain (the chromodomain) and an SNF2-related helicase/ATPase domain [19]. Thus, in future it may be of interest to study *CHD2* gene deletions also for presence of mutations in this gene and also to screen ALL patients in general for *CHD2* gene mutations.

Besides, duplication of *DCC* gene in 18q21.2 was present in 3 of the 34 (9 %) studied cases. *DCC* is a member of the immunoglobulin superfamily of cell adhesion molecules and acts as a transmembrane dependence receptor for netrins, key factors in the regulation of axon guidance during development of the central nerve system. Amplification of *DCC* gene was previously reported in chronic lymphocytic leukemia (CLL) [20, 21], however, this is the first report for *DCC* gene amplification in ALL. To evaluate the role of the *DCC* gene and to elaborate its potential as a molecular marker in ALL still needs more studies.

In general, submicroscopic CNAs were identified most frequently in chromosomes #7 and #9. CNAs in #7 involved deletion of *IKZF1* at 7p12.2 that encodes IKAROS protein and is required for the development of all lymphoid lineages in 5 of 34 (14 %) studied CN-ALL cases. According to the literature deletions and/or sequence mutations of *IKZF1* are present in 15 % of pediatric B-ALL, including ~70 % of BCR-ABL–positive ALL and with high-risk of relapse ~30 % of BCR-ABL–negative B-ALL [22]. However, deletions of *IKZF1* are predominantly monoallelic and involve the N-terminal zinc-finger domain of IKAROS protein and result in expression of dominant-negative isoforms with cytoplasmic localization and oncogenic activity as well as an association with very poor outcome [23, 24]. Thus, *IKZF1* has newly been considered as a prognostic marker for B-ALL and might be useful for risk stratification [24, 25].

Cyclin dependent kinase 6 (*CDK6*) at 7q21.2, is the catalytic subunit of a protein kinase complex that regulates cell cycle G1 phase progression and G1/S transition. Deletion of *CDK6* was identified in this study in 4 of 34 (12 %) of ALL cases. It has been shown recently that inhibition of CDK6 may lead to overcome the differentiation block seen in acute myelogenous leukemia (AML) with *MLL* translocations [26]. Further studied for this gene may also be recommended for better understanding of ALL biology.

**Figure 3:**
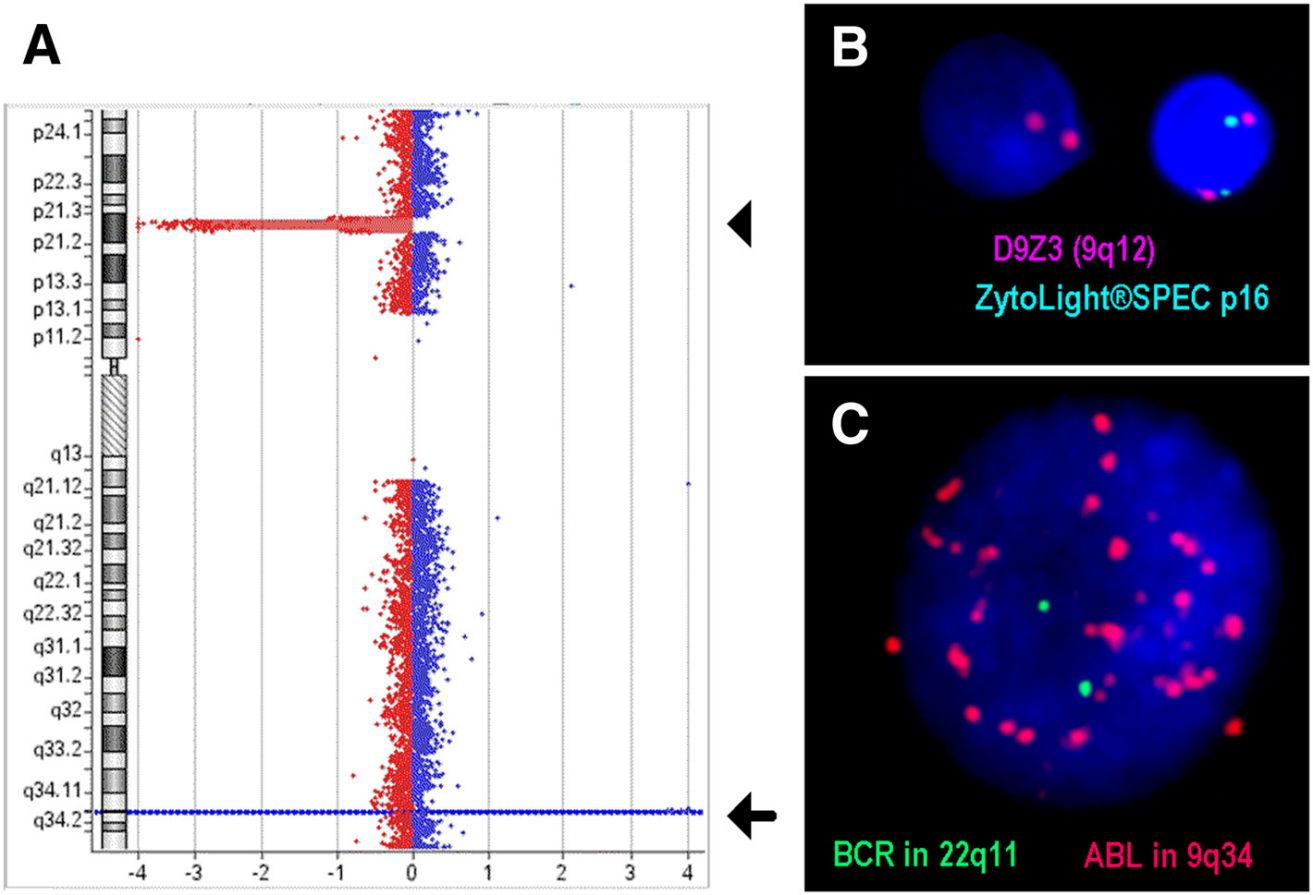
aCGH from case Nr. P61 showed two CNAs in chromosome 9; at 9p21.3 a homozygous deletion (arrowhead) and at 9q34.12 to 9q34.13 an amplification (arrow). **a** FISH confirmed presence of the homozygous deletion in 9p21.3 in interphase. **b** An amplification present as double minutes was confirmed using a probe specific for the *ABL*-gene

The majority of #9 abnormalities is involving deletions of cell cycle regulatory genes at 9p21.3. The main target to deletions is *CDKN2A* which encodes for the two transcripts *p16/INK4A* and *p14/ARF* (alternative splicing), followed by *CDKN2B* gene (*p15/INK4B*); both are tumor suppressor genes. Deletions of *CDKN2A/B* can be found in 30 and 50 % of B-ALL and T-ALL cases, respectively [23, 25, 27]. In the present study such deletions were only found in 8/34 (24 %) of the studied ALL cases, which is most likely due to low case numbers. *CDKN2A*/B deletion can be detected at initial diagnosis or acquired at relapse, suggesting that *CDKN2A/B* deletion is a secondary genetic event. Also, the outcome of cases with *CDKN2A/B* deletion depends on the status of the second allele, as homozygous deletions are associated with poor outcome and heterozygous deletions represent markers for favorable outcomes [27, 28]. T-ALL-case P61 had such a prognostically adverse homozygous deletion in 9p21.3 together with amplification of 9q34.12 to 9q34.13; the latter contains the *ABL1* and *NUP214* genes (Fig. 3). *NUP214-ABL1* fusion gene amplification was previously mainly observed in T-ALL and associated with poor outcome [6].

Another recurrent deletion in #9 in the studied ALL cases involved the *PAX5* gene located in 9p13.2, which encodes for a protein with key roles in lymphoid development. It was found to be deleted in B-ALL (*n* = 2) and T-ALL (*n* = 1 showed short arm 9p deleted) in this study. In the literature, deletion of *PAX5* was reported in 31.7 % of B-ALL and also it has been involved in several chromosomal translocations [29, 30]. In a recent report, *PAX5* deletion was observed in only 10 % and 18 % in children and adult B-ALL, respectively; notably *PAX5* deletion was frequently accompanied by deletion of *CDKN2A* (83.3 % of children and 100.0 % of adults) [28]. Also *PAX5* was found to be a common target in leukemogenesis of B-ALL, but not associated with adverse outcome [15]. In future, *PAX5* could be used as one of the molecular markers in diagnosis and monitoring of the disease, especially in B-ALL [28–30].

Besides, other CNAs have been identified here, encompassing single or few genes, only. Many of CN losses involve cell cycle regulatory and/or putative tumor suppressor genes like 10q23.3 (*PTEN*; *n* = 6), 13q14.2 (*RB1*; *n* = 3), and 17p13.1 (*TP53*; *n* = 4), or transcriptional regulators and co-activators like 3q26.32 (*TBL1XR1*; *n* = 1), 12p13.2 (*ETV6*; *n* = 2), 21q22.12 (*RUNX1*; n = 1) and 21q22.2 (*ERG*; *n* = 2), or regulators of chromatin structure and epigenetic regulators like 16p13.3 (*CREBBP*; *n* = 2). Although, oncogene overexpression resulting from gene duplication is infrequent in ALL, we found *MYB* duplication in one case, too. These observations of gene loss of function or overexpression being involved in leukemic transformation [15, 31] underline the heterogeneity of different ALL cases and the potential of molecular approaches to identify new subgroups of this disease.

The present study also highlights, that most likely all CN-ALL cases hold cryptic genomic alterations. DNA sequencing and single-nucleotide polymorphism (SNP) arrays have been used to detect mutations for a number of target genes that are known to key roles in lymphoid development. Thus, somatic mutations have been identified in both B and T-ALL patients [2]. For instance, mutations in *JAK2* were identified in 10 % of high-risk childhood B-ALL and shown to be associated frequently with other abnormalities, including deletions or mutations of *IKZF1* and overexpression the *CRLF2* gene [23]. In T-ALL, *NOTCH1*-activating gene mutation has been found in 60 % and *FBXW7-*inactivating gene mutation occurs in 20 % of pediatric T-ALL [32]. Less commonly, mutations in *PTEN*, *WT1*, amplification of *MYB* and sequence mutations in RAS signaling (*NRAS*, *KRAS*, and *NF1*) and tumor suppression (*TP53*) have been identified in ALL [8, 31].

Overall, sensitive methods to detect cryptic chromosomal aberrations in CN-ALL are useful and necessary for genetic risk–based classification and correct determination of treatment protocols. The present study highlights that molecular cytogenetic approaches together with molecular methods are suited to identify cryptic rearrangements and potential target genes that involved in leukemogenesis and progression of the disease. Also it could be  demonstrated that aCGH is a highly efficient tool for detection of CNAs in CN-ALL. However, while aCGH (and MLPA) provide data on imbalanced genomic alterations, (molecular) cytogenetics additionally detects different leukemic subclones within one sample, as well as balanced translocations leading to tumor-specific fusion genes. It seems to be valid, that there is no leukemia clone without genetic alterations; we just have to use the appropriate techniques to identify them. In conclusion, to obtain a comprehensive picture of all relevant changes in each individual ALL case data from cytogenetics, FISH, MLPA and aCGH needs to be considered and included in diagnostics; however, sometimes such investigations may be hampered by lack of sufficient cellular material, as also in this study, where only 34/61 cases could also be studied on DNA level or other previous studies [16, 33].

## Methods

### Patients and sample preparation

Cell suspensions were obtained from bone marrow collected from 61 patients diagnosed with ALL (31 with B-ALL, 12 with T-ALL and 18 with undefined ALL; Additional file 1: Table S1). The samples were obtained under informed consent of the corresponding patients and according to institutional ethical committee guidelines (ethical commission of the university clinic Jena, Germany; code 1105-04/03).

### GTG-banding

The bone marrow cells were unstimulated cultivated for 24 hours (with and without colchicin) and 48  h, and a standard cytogenetic cell preparation following air drying method was done [34]. GTG-banding was routinely done in each sample following standard procedures. Twenty metaphases were obtained for cytogenetic evolution on a banding level of 250–300 bands per haploid karyotype [35]. Apart from 4 all 61 studied cases had a normal karyotype of 46,XX or 46,XY. In one case the karyotype could not be determined due to low metaphase quality; one case just had (most likely age associated) loss of an X-chromosome in a subset of the cells, one case had a questionable der(19) in all cells, and another one a trisomy 14 in 6/20 studied cells.

### Molecular cytogenetics

Fluorescence *in situ* hybridization was done according to standard procedures and/or according to manufacturer’s instructions.

Homemade were the following probes and probe sets:

- 24-color-FISH using all human whole chromosome painting (WCP) probes [36];
- FISH-banding probe-sets as follows: genome wide multitude multicolor banding (mMCB) and chromosome specific high resolution array-proven multicolor-banding (aMCB) [16, 37, 38];
- WCP probes for all chromosomes were homemade [36].
- The following commercially available locus-specific probes (LSPs) (Additional file 2: Table S2) were used to validate and possibly confirm the breakpoints found in mMCB, aCGH and/or MLPA: from Abbott/Vysis (Wiesbaden, Germany), Kreatech Diagnostics (Amsterdam, Netherland), ZytoVision (Bremerhaven, Germany), and DNA from bacterial artificial chromosome (BACs) probes obtained from Resources Center (Oakland, USA) were labeled by PCR with SpectrumGreen, SpectrumOrange or TexasRed-dUTP and applied in two- or three-color FISH-approaches. For each interphase FISH analysis to determine the percentage of specific aberrations, at least 200 interphase nuclei were examined per sample and FISH-probe – the applied probes can be found in Additional file 2: Table S2.
- Homemade and previously reported chromosome-specific sub-CTM- (= subtelomere -/ subcentromere oriented) probe-sets were applied in selected cases [13] (Additional file 1: Table S1).

### DNA isolation

Genomic DNA was extracted from cells fixed in acetic acid-methonal (1:3) by Puregene DNA Purification Kit (Gentra Systems, Minneapolis, MN, USA). DNA concentration was determined by a Nanodrop spectrophotometer. The quality of DNA was checked using agarose gel electrophoresis. DNA-samples extracted from fixed cells of 2 healthy males and 2 healthy females by the same method were used as reference samples.

### MLPA analysis

SALSA MLPA P377-A1 Hematologic malignancies probemix was used for this study (MRC- Holland, Amsterdam, The Netherlands). This probemix contains probes for 37 genes covered by 54 probes, which have diagnostic or prognostic significant role in hematologic malignancies. MLPA was performed according to the manufacturer’s protocol, which includes three reaction phases: hybridization, ligation, and PCR amplification. Amplified probes and GeneScan LIZ 500 (Applied Biosystems, Foster City, USA) standard were separated by capillary electrophoresis using a ABI-PRISM 3130XL Genetic Analyzer (Applied Biosystems, Foster City, USA). GeneMarker (SoftGenetics, USA) was used to analyzeMLPA data. Detection threshold was set at 0.65-1.35; control samples of four healthy donors were included in each run.

### Array-comparative genomic Hybridization (aCGH)

aCGH was performed using Agilent SurePrint G3 Human Genome microarray 180 K (Agilent Technologies, Santa Clara, CA, USA), an oligonucleotide microarray containing 170,334 probes 60-mer with a ~13 kb overall median probe spacing (11 kb in Refseq-genes). Genomic DNA of patients was co-hybridized with a sex-mismatched control DNA (G1471 or G1521; Promega, Mannheim, Germany). Labeling was performed using Agilent Genomic DNA enzymatic labeling kit (Agilent) according to the manufacturers’ instructions. After hybridization and washing, the aCGH slide was scanned on an Agilent scanner, processed with Feature Extraction software (v12.0.2.2) and results were analyzed using Cytogenomics (v3.0) using ADM2 as aberration algorithm.

## Additional files

Additional file 1: Table S1. All 61 CN-ALL cases studied; for each case age, gender and subtype of ALL is given. Also all FISH-probes, probe sets and approaches applied for each case are listed. Abbreviations: n.d. = not determined, y = year. Additional file 2: Table S2. List of locus specific probes used in the present study for further characterization of acquired aberrations and/or determination of the percentage of deletions or duplications as determined by aCGH or MLPA.

## Abbreviations

aCGH: Array-comparative genomic hybridization
ALL: Acute lymphoblastic leukemia
aMCB: Array-proven multicolor-banding
AML: Acute myelogenous leukemia
BAC: Bacterial artificial chromosome
B-ALL: B-cell ALL
Bp: Basepairs
CLL: Chronic lymphocytic leukemia
CN: Copy number
CNA: Copy number alteration
CN-ALL: ALL according to banding cytogenetics with normal karyotype
CNVs: Copy number variations
DNA: Deoxyribonucleic acid
FISH: Fluorescence *in situ* hybridization
GTG: G-banding with trypsin-Giemsa
LSPs: Locus-specific probes
MLPA: Multiplex ligation-dependent probe amplification
mMCB: Multitude multicolor banding
n.d.: Not determined
PCR: Polymerase chain reaction
SNP: Single-nucleotide polymorphism
sub-CTM: Subtelomere -/ subcentromere oriented
T-ALL: T-cell ALL
TPA: 2-O-tetradecanoylphorbol-13-acetate
WCP: Whole chromosome painting
Y: Year
