# Supplementary material for: High rates of submicroscopic aberrations in karyotypically normal acute lymphoblastic leukemia
Source: Mol Cytogenet. 2015 Jun 30;8:45. doi: 10.1186/s13039-015-0153-4 (PMC4486437; doi:10.1186/s13039-015-0153-4)
Supplement: Additional file 1: Table S1. — All 61 CN-ALL cases studied; for each case age, gender and subtype of ALL is given. Also all FISH-probes, probe sets and approaches applied for each case are listed. Abbreviations: n.d. = not determined, y = year. [file 13039_2015_153_MOESM1_ESM.docx]

**Supplementary Table 1**

| **case number** | **age [y]** | **gender** | **banding cytogenetic result** | **FISH probes** | **molecular approaches** |
| --- | --- | --- | --- | --- | --- |
| **B-ALLs** | | | | | |
| P1 | 1 | F | 46,XX[7] | mMCB | MLPA  aCGH |
| P8 | 30 | M | 46,XY[8] | mMCB  LSPs #21 | MLPA |
| P13 | 34 | M | 46,XY[8] | mMCB  LSPs #10, #17 | MLPA  aCGH |
| P14 | 18 | M | 46,XY[20] | mMCB | n.d. |
| P17 | 27 |  | 46,XX[7] | mMCB | aCGH |
| P22 | 42 | F | 46,XX[20] | mMCB | n.d. |
| P23 | 59 | F | 46,XX[14]/47,XX,+14[6] | mMCB  MCB#14  LSPs #14 | MLPA  aCGH |
| P25 | 71 | F | 46,XX[5] | mMCB | n.d. |
| P28 | 84 | M | 46,XY[5] | mMCB  MCB#11  LSPs #9, #11 | MLPA  aCGH |
| P29 | 59 | M | 46,XY[5] | mMCB | n.d. |
| P37 | 52 | M | 46,XY[5] | mMCB | n.d. |
| P40 | 57 | F | 46,XX[6] | mMCB  MCB #11 | n.d. |
| P41 | 31 | M | 46,XY[4] | mMCB  MCB #15  WCP #8 | n.d. |
| P43 | 69 | F | 46,XX[20] | mMCB  MCB #11  CEP #4  LSPs #11 | MLPA  aCGH |
| P44 | 24 | M | 46,XY[3] | mMCB  WCP #4, #10 | n.d. |
| P48 | 39 | M | 46,XY[20] | mMCB  M-FISH  MCB #6, #11  WCP #6, #11  subCTM11  LSPs #6, #11 | aCGH |
| P49 | 39 | F | 46,XX[10] | mMCB | aCGH |
| P50 | 21 | F | 46,XX[2] | mMCB | n.d. |
| P51 | 59 | F | 46,XX[6] | mMCB | MLPA  aCGH |
| P52 | 21 | M | 46,XY[4] | mMCB | MLPA  aCGH |
| P53 | 34 | M | 46,XY[5] | mMCB | MLPA  aCGH |
| P55 | 19 | M | 46,XY[6] | mMCB | MLPA  aCGH |
| P56 | 47 | M | 46,XY[20] | mMCB | MLPA  aCGH |
| P57 | 56 | M | 46,XY[3] | mMCB | MLPA  aCGH |
| P58 | 20 | F | 46,XX[20] | mMCB  MCB #14  WCP #8, #14  LSPs #9, #14 | MLPA  aCGH |
| P59 | 25 | M | 46,XY[2] | mMCB | n.d. |
| P62 | 34 | F | 46,XX[3] | mMCB | n.d. |
| P64 | 4 | F | 46,XX,?der(19)[20] | mMCB  MCB #5, #9, #16, #19  WCP#5, #9, #16, #19, X  LSPs #5, #9, #16, #19 | aCGH |
| P65 | 18 | M | 46,XY[10] | mMCB  MCB #8, #14,  LSPs #8, #14 | n.d. |
| P66 | 0.5 | F | n.d. | M-FISH  MCB #10, #11; #14; WCP #10, #11, #14, LSPs #10, #11, #14 | aCGH |
| P67 | 12 | M | 46,XY[15] | M-FISH  MCB #1, #7  LSPs #1, #7, #11 | n.d. |
| **T-ALLs** | | | | |  |
| P3 | 19 | M | 46,XY[8] | mMCB | n.d. |
| P5 | 22 | F | 46,XX[12] | mMCB | MLPA  aCGH |
| P6 | 16 | M | 46,XY[9] | mMCB  M-FISH  MCB #3, #5, #10  WCP #4 | MLPA  aCGH |
| P7 | 26 | M | 46,XY[7] | mMCB  M-FISH  MCB #2, #9, #11, #18  WCP #10, #14  subCTM #11  LSPs #2, #9, #18 | MLPA  aCGH |
| P15 | 44 | F | 46,XX[5] | mMCB | n.d. |
| P18 | 36 | M | 46,XY[5] | mMCB  MCB5  LSPs #18 | MLPA |
| P26 | 28 | F | 46,XX[5] | mMCB | n.d. |
| P32 | 27 | M | 46,XX[17] | mMCB  MCB #6, #10, #14  subCTM #6  LSPs #9, #12, #13 | MLPA |
| P35 | 40 | M | 46,XY[10] | mMCB  LSPs #9 | MLPA  aCGH |
| P36 | 58 | M | 46,XY[20] | mMCB | n.d. |
| P38 | 22 | M | 46,XY[3] | mMCB | MLPA  aCGH |
| P61 | 18 | F | 46,XX[20] | mMCB  M-FISH  MCB #2, #4;#7, #10  WCP #2, #7, #10  LSPs #2;#7, #10 | MLPA  aCGH |
| **B- or T ALLs (not clinically well defined)** | | | | |  |
| P2 | 23 | F | 46,XX[11] | mMCB | n.d. |
| P4 | 18 | F | 46,XX[2] | mMCB | n.d. |
| P9 | 4 | F | 46,XX[2] | mMCB | n.d. |
| P10 | 15 | F | 46,XX[5] | mMCB | n.d. |
| P11 | 26 | M | 46,XY[8] | mMCB  WCP #11, #22 | aCGH |
| P12 | 24 | F | 46,XX [5] | mMCB | n.d. |
| P16 | 17 | F | 46,XX[7] | mMCB  LSPs #9; #12 | MLPA  aCGH |
| P19 | 9 | M | 46,XY[5] | mMCB | n.d. |
| P21 | 62 | M | 46,XY[11] | mMCB | aCGH |
| P24 | 23 | M | 46,XY[12] | mMCB  LSPs #18 | MLPA |
| P27 | 71 | F | 46,XX[5] | mMCB | n.d. |
| P30 | 46 | M | 46,XY[6] | mMCB  MCB #9 | MLPA |
| P31 | 58 | M | 46,XY[5] | mMCB | n.d. |
| P33 | 76 | F | 46,XX[4]/45,X,-X[14] | mMCB  LSPs #9, #12, #18 | MLPA  aCGH |
| P34 | 61 | M | 46,XY[7] | mMCB  MCB5  LSPs #5 | n.d. |
| P39 | 52 | F | 46,XX[5] | mMCB | n.d |
| P46 | 63 | M | 46,XY[8] | mMCB  CEP #7  WCP#5, #10 | MLPA  aCGH |
| P47 | 59 | M | 46,XX[6] | mMCB | MLPA  aCGH |
