# Supplementary material for: High rates of submicroscopic aberrations in karyotypically normal acute lymphoblastic leukemia
Source: Mol Cytogenet. 2015 Jun 30;8:45. doi: 10.1186/s13039-015-0153-4 (PMC4486437; doi:10.1186/s13039-015-0153-4)
Supplement: Additional file 2: Table S2. — List of locus specific probes used in the present study for further characterization of acquired aberrations and/or determination of the percentage of deletions or duplications as determined by aCGH or MLPA. [file 13039_2015_153_MOESM2_ESM.docx]

**Supplementary Table 2**

| **probe** | **locus** |
| --- | --- |
| CEB108/T7 (Abbott/Vysis) | 1p36.3 |
| ZytoLight®SPEC *ALK* (ZytoVision) | 2p23.2~23.1 |
| D2S447 (Abbott/Vysis) | 2q37.3 |
| ZytoLight®SPEC *TFG* (ZytoVision) | 3q12.2 |
| RP11-114M1 and RP11-91K9 (*TBL1XR1*) | 3q26.32 |
| D3S4559 (Abbott/Vysis) | 3p26.3 |
| CEP4 = D4Z1 (Abbott/Vysis) | 4p11-q11 |
| C84c11/T3 (Abbott/Vysis) | 5p15.33 |
| LSI D5S721 (Abbott/Vysis) | 5p15.2 |
| LSI *EGR1*/D5S23 (Abbott/Vysis) | 5q31 |
| POSEIDON *PDGFRB* (Kreatech) | 5q33 |
| D5S2907 (Abbott/Vysis) | 5q35.3 |
| ZytoLight®SPEC *MYB* (ZytoVision) | 6q23.2~q23.3 |
| ZytoLight®SPEC CEN6 = D6Z1 (ZytoVision) | 6p11.1-q11.1 |
| ZytoLight®SPEC *ESR1* (ZytoVision) | 6q25.1 |
| RP11-112P10 (*RELN*) | 7q22.1 |
| VIJyRM2000 (Abbott/Vysis) | 7q36.3 |
| ZytoLight®SPEC *CDKN2A* (ZytoVision) | 9p21.3 |
| ZytoLight®SPEC CEN9 = D9Z3 (ZytoVision) | 9q12 |
| LSI *ABL* (Abbott/Vysis) | 9q34 |
| Z96139 (Abbott/Vysis) | 10p15.3 |
| ZytoLight®SPEC *WT1* (ZytoVision) | 10p13 |
| ZytoLight®SPEC CEN 10 = D10Z1 (ZytoVision) | 10p11.1-q11.1 |
| ZytoLight®SPEC *PTEN* (ZytoVision) | 10q23.3 |
| ZytoLight®SPEC *FGFR2* (ZytoVision) | 10q26.13 |
| D10S2290 (Abbott/Vysis) | 10q26.3 |
| D11S2071 (Abbott/Vysis) | 11p15.5 |
| POSEIDON NUP98 (Kreatech) | 11p15.4 |
| ZytoLight®SPEC *BIRC3* (ZytoVision) | 11q22.2 |
| ZytoLight®SPEC *ATM* (ZytoVision) | 11q22.3 |
| LSI *MLL* (Abbott/Vysis) or POSEIDON *MLL* (Kreatech) | 11q23.3 |
| D11S1037 (Abbott/Vysis) | 11q25 |
| 8M16/SP6 (Abbott/Vysis) | 12p13.3 |
| ZytoLight®SPEC ETV6 (ZytoVision) | 12p13.2 |
| LSI 13 (*RB1*) (Abbott/Vysis) | 13q14.2 |
| LSI D13S25 (Abbott/Vysis) | 13q14.3 |
| LSI *IGH* (Abbott/Vysis) | 14q32.33 |
| D14S1420 (Abbott/Vysis) | 14q32.33 |
| ZytoLight®SPEC *FUS* (ZytoVision) | 16p11.2 |
| ZytoLight®SPEC TP53 (ZytoVision) or LSI p53 (Abbott/Vysis) | 17p13.1 |
| CEP 18 = D18Z1 (Abbott/Vysis) | 18p11.1-q11.1 |
| LSI *BCL2* (Abbott/Vysis) | 18q21 |
| RP11-346H17 (*DCC*) | 18q21.2 |
| ZytoLight®SPEC *MALT1* (ZytoVision) | 18q21.32 |
| ZytoLight®SPEC 19q13 (ZytoVision) | 19q13.3 |
| POSEIDON *MLLT1* (Kreatech) | 19p13.3 |
| ZytoLight®SPEC 19p13 (ZytoVision) | 19q13.43 |
| ZytoLight®SPEC *RUNX1* (ZytoVision) | 21q22.12 |
| ZytoLight®SPEC *ERG* (ZytoVision) | 21q12.13 |
| LSI BCR (Abbott/Vysis) | 22q11.2 |
